# Dependence of Creep Strain and Fatigue Behavior on Surface Characteristics of Resistive Strain Gauges

**DOI:** 10.3390/mi13030379

**Published:** 2022-02-26

**Authors:** Yinming Zhao, Siyang Tan, Chaofan Zhang, Yang Liu, Linglu Wang, Yongqian Li, Qun Hao

**Affiliations:** 1 Beijing Changcheng Institute of Metrology & Measurement, Beijing 100095, China; zhaoyinming@cimm.com.cn (Y.Z.); wanglinglu@cimm.com.cn (L.W.); 2 Key Laboratory of Micro/Nano Systems for Aerospace of Ministry of Education, Northwestern Polytechnical University, Xi’an 710072, China; sytan@mail.nwpu.edu.cn (S.T.); cfzhang@mail.nwpu.edu.cn (C.Z.); ly201169@mail.nwpu.edu.cn (Y.L.); 3 School of Optics and Photonics, Beijing Institute of Technology, Beijing 100081, China; qhao@bit.edu.cn

**Keywords:** resistive strain gauge, surface roughness, creep behavior, fatigue life, force sensor, wet etching, resistance trimming, roughness, sensitive grids, textures

## Abstract

Creep behavior and fatigue life are important performance indexes that affect the long-term stability of resistive strain gauges. The resistive strain gauges, fabricated with wet etching and resistance trimming, present micro-morphology such as textures and uneven edges on the surface and side-wall profile of sensitive grids. This paper observed the micro-morphology of the sensitive grids by microscope and analyzed its range of geometric dimensions. A sine function was used to establish equivalent geometric models for the surface textures and side-wall profile. Based on time hardening theory and the S–N curve, the dependence of micro-morphology of metal resistive strain gauges on creep behavior and fatigue life was studied. The results indicate that the roughness of micro-morphology has an influence on creep behavior and fatigue life. The surface textures and side-wall profile lead to the increase of creep strain and the decrease of fatigue life in varying degrees. When 60% of the ultimate stress of the strain gauges is loaded, the average creep strain in steady-state calculated by the maximum roughness of the side-wall profile reaches up to 6.95 times that of the perfect flat surface. Under the condition of loading 70% of the ultimate stress and the same roughness, the fatigue life led by side-wall profile could be reduced to 1/25 of the textured surface. The obtained achievements promote an understanding for optimizing the fabrication process of resistive strain gauges as well as developing high-precision and long-life force sensors.

## 1. Introduction

Both wet etching and resistance trimming are critical procedures in the fabrication process of foil-type strain gauges. For the strain gauge fabricated by wet etching, there will be uneven micro-morphology on the sidewall of sensitive grids, which is specifically manifested as irregular transition profile [1,2]. In addition, the process of wet etching cannot precisely control the dimensional size of sensitive grids. Consequently, resistance trimming is needed after wet etching since the resistance value usually deviates from the standard ones [1]. Two methods are generally used in resistance trimming, one is chemical corrosion and the other is mechanical polishing. The former uses ferric chloride to corrode sensitive grids surface to reduce the thickness of strain gauges, while the latter uses abrasives to polish the surface directly [1]. However, both will leave uneven textures on the surface.

In working conditions, these uneven side-wall profile and textures cause stress concentrations to appear within the micro-morphology zone [3], which will worsen the creep behavior and working lifetime of strain gauges [4,5]. By using sine hyperbolic geometrical model, three-dimensional notches on nickel-based alloy specimen were studied, and quantitative research found that plastic deformation resulted in fracture at the root of notches when stress concentrations reached the yield strength of the material [6]. As strain gauges are subjected to constant loads, the different contour shapes and dimensional profiles greatly affect their creep damage distribution and working life [7]. Geometric shapes such as radius and angles of notches will lead to local stress concentrations, which affect fatigue life [6] and creep damage [7]. Based on the fatigue crack growth model, it was found that the maximum valley depth on the surface can act as a defect size to characterize and predict the fatigue life of mechanical parts [8]. In addition, experimental results have revealed that the notch geometry affects the fatigue life of materials [9]. In addition, the orthogonal experiment shows that the diameter, length, and interval of sensitive grids play a conspicuous role in the fatigue life of strain gauges [10]. Other factors affecting creep behavior and fatigue life include structural size, surface prestress, working environment, and loads [11].

From the perspective of mechanism and material science, the above work studied the influence of notch shapes and geometry sizes on fatigue life, and the dependence of inner microscopic stress on creep behavior. However, as we know by now, the correlations between micro-morphology features and the creep behavior and fatigue life of strain gauges have not been considered in issued literature. In this paper, roughness models based on the observed micro-morphology were established to investigate the creep behavior and fatigue life of strain gauges. Here, the micro-morphology refers to both the upper surface textures and side-wall profile of metal sensitive grids. Two surface textures are modeled, and they are longitudinal and transverse, respectively.

## 2. Observation and Analysis Procedures

**Figure 1:**
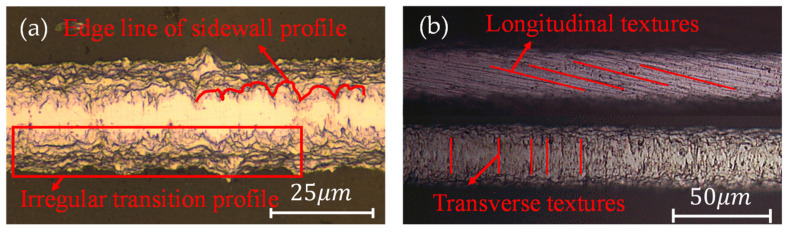
Upper surface roughness and side-wall profile of sensitive grids. (**a**) optical image of sensitive grids and one magnified strip showing the rough side-wall profile; (**b**) upper surface of sensitive grids observed in a microscope.

By observing dozens of strain gauge samples, we obtained close-up pictures of the micro-morphology on sensitive grids. In Figure 1, we have randomly selected several observation photos as representatives to illustrate the problem. The light-colored area indicates the sensitive grids, and the dark-colored area is the gap between them. Since the images were taken from a top view, the dark area is actually where the adhesive layer lies, and further down is the strain gauge substrate. The side-wall profile of sensitive grids caused by wet etching is complex. In Figure 1a, the irregular transition profile is presented on the edge. The transition area can be regarded as a superposition of multi-layer contours. In contrast, the surface textures of the sensitive grids are more regular [2]. Depending on different orientations of the resistance trimming, the direction of these textures can be sorted into parallel and perpendicular to the length direction of sensitive grids. Figure 1b shows that the mechanical polishing process dominates the contribution to the textures on sensitive grid surfaces. The essential textures seem to be approximate with resemblance.

**Figure 2:**
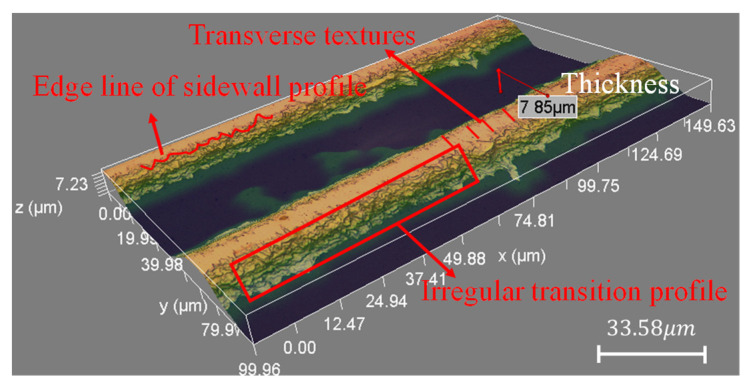
3D model of a single grid measured by a Zego interferometer.

To have a more intuitive visual understanding, three-dimensional micro-morphology of the selected sensitive grid sample was pictured by an optical microscope (Figure 2). From this perspective, there are obvious traces left on the surface after resistance trimming, and the green area shows a transition profile of hilly edge. Additionally, the thickness of the sensitive grid decreases from the center to the edge, and the discrete spots caused by wet etching emerged at the root.

**Figure 3:**
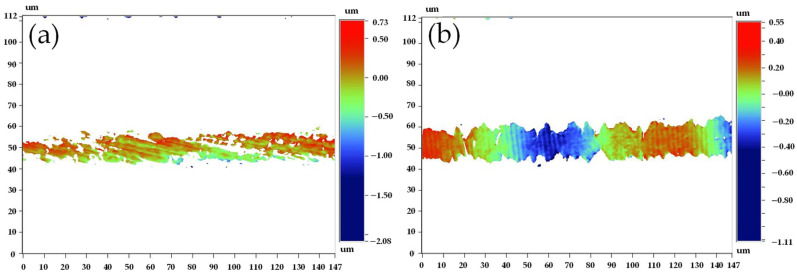
Spectral distribution of the (**a**) longitudinal texture and (**b**) transverse texture roughness obtained by optical profilometer measurements. The side-wall profile of sensitive grid can also be observed in (**b**).

Using an optical profilometer, we measured the geometrical dimensions of the micro-morphology for all samples, and the representative results are shown in Figure 3. Figure 3a shows the optical image obtained from the measurement of the longitudinal textures on the surface of a single grid wire, while Figure 3b shows the transverse ones. It is easy to see that, for a single wire, whether longitudinal or transverse, the spectrum characterizing the texture roughness has a regular distribution and the size fluctuates slightly in a certain range. This variation is similar to a sine function [11]. We averaged the peaks of these values to represent the surface texture roughness of the corresponding grid wire and conducted the same treatment for all samples. Through statistics, we found that the variation range of the texture roughness is less than 0.4 µm. Still, it is worth noting that the optical image in Figure 3b could also show the pattern of the side-wall profile, and the approximation of the sine function to the profiles is also applicable [11]. This conclusion is also supported by observing the wavy edge line closest to the upper surface in Figure 1a (as shown by the red curve). Adopting image processing technology to analyze and through the statistics of all samples, we calculated the maximum roughness of the sidewall is about 4 µm.

**Figure 4:**
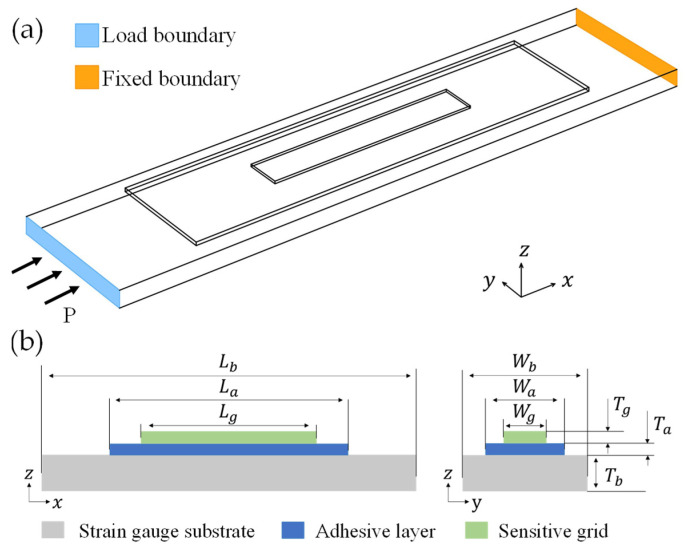
Geometrical model (**a**) and schematic cross section (**b**) of sensitive grids for the simulation calculations. The boundary conditions and the geometrical dimensional sizes are marked in the drawings.

According to the structure of strain gauge samples, a three-layer simulation model was adopted in Figure 4a in order to study the fatigue life and creep behavior. This model consists of polyimide substrate, epoxy resin adhesive layer, and sensitive grid layer of constantan alloy, the basic parameters of these three materials used in this study are shown in Table 1 [12,13]. There are two boundaries on the substrate, one was set to be fixed and the other was a load one. The stress was input to the load boundary in creep and fatigue simulation. Particularly, to highlight the research priority, we simplified the sensitive grid into a single wire (Figure 4), and dimensions of the entire model refer to the geometric parameters of the samples [13,14], the specific data are shown in Table 2.

**Figure 5:**
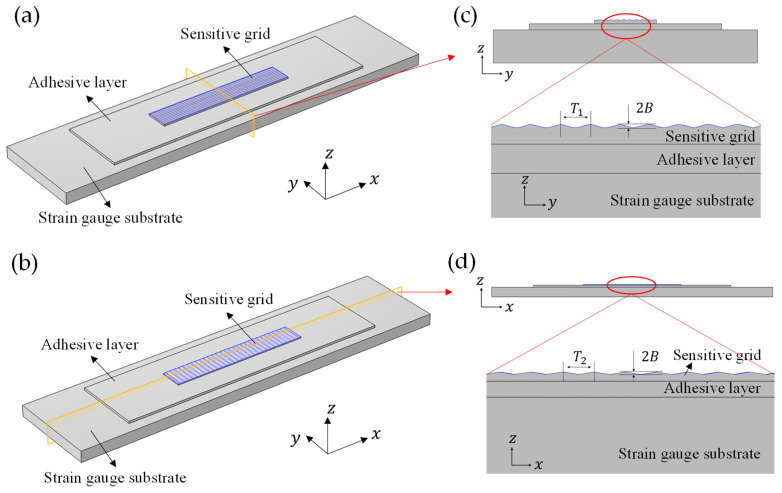
Schematic drawing of longitudinal (**a**) and transverse (**c**) surface textures in sensitive grids. The dimensional schematic of the sinusoidal shape in the cross section view along the longitudinal (**b**) and transverse (**d**) direction.

**Figure 6:**
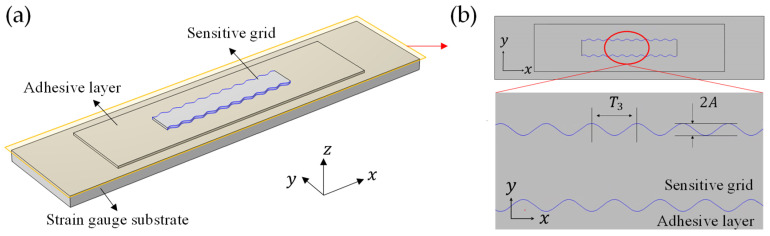
Schematic diagram of the side-wall profile model (**a**) and its crossed section view (**b**).

In this paper, the longitudinal (*x*-direction) and transverse (*y*-direction) sinusoidal geometric models shown in Figure 5a,b were modeled to simulate the actual surface textures on sensitive grids (Figure 1b). Close-up views of the sine functions are shown in Figure 5c,d, the amplitude B (0–0.4 µm) represents the roughness of textures, and the periods $T_{1}$, $T_{2}$ are representations for the texture density. Similarly, the sinusoidal geometric model for the side-wall profile was established in Figure 6, the amplitude A (0–4 µm) represents the roughness of the side-wall profile, and $T_{3}$ is the representation for profile density. In this study, $B/T_{g}$ and $A/W_{g}$ are used to represent the amplitude variation of textures and side-wall profile roughness. $T_{1}$, $T_{2}$ and $T_{3}$ takes 6.28/5, 6.28/10 and 6.28/30 µm, respectively, according to the commercial strain gauges we observed.

**Figure 7:**
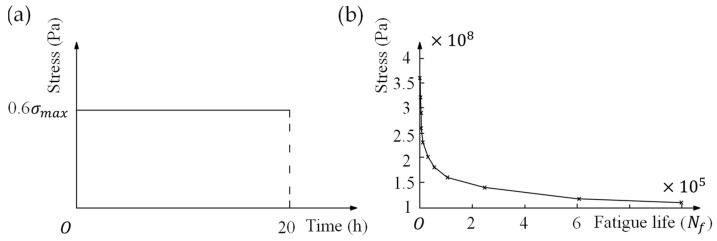
(**a**) Step load applied for the creep simulation; (**b**) S–N curve of alloy steel used.

The creep strain we studied refers to the one only caused by a creep effect, and it is defined as the difference between total and elastic strain under the external force. The creep strain of a material is mainly related to time, temperature, and stress [15]. Norton–Bailey time hardening theory assumes that, under the condition of constant load and temperature, creep strain is a function of time but no relevance with other factors. Strain exponential equation based on this assumption is [16]:

(1) $$\varepsilon_{c}=C\sigma^{n}t^{m},$$

where parameters C, n, and m are constants, which refer to creep rate coefficient, stress exponent, and time hardening exponent respectively, and they depend on the nature of materials [11]. The values of these constants for constantan alloy in this analysis are shown in Table 3 [17]. Creep simulation is carried out under a constant load as shown in Figure 7a. The load value was 60% of the ultimate stress of the strain gauge, and the loading time was 20 h.

The fatigue curve (also called S–N curve) describes correlations between the value of stress $\sigma_{a}$ and the number of cycles $N_{f}$ when fatigue damages occur in metal structures [18,19]. During fatigue simulation, the S–N curve of alloy steel shown in Figure 7b was selected as the reference criteria. For the fatigue life of strain gauges, which is commonly measured under uniaxial cyclic stress, we set the cyclic stress ratio to 0 in order to fit the reality. In this case, the fatigue life obtained is the maximum one. Under different loads, by calculating the maximum stress value in the stress distribution of the models, we can obtain the fatigue life for corresponding load through S–N curve.

Moreover, for creep and fatigue are both dynamic processes, transient was set as the research object in all simulations. Please see supplementary material for the detailed theoretical description of creep and fatigue life [11,15,16,18,20,21,22,23,24].

## 3. Results and Discussion

### 3.1. Creep Strain

**Figure 8:**
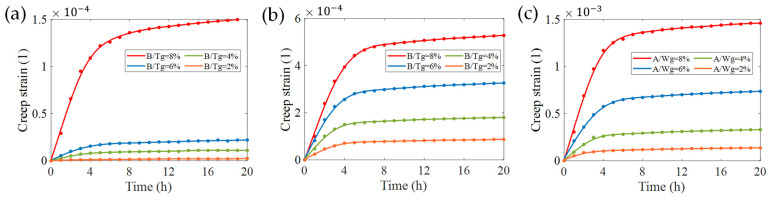
Creep strain of sensitive grids with varied surface roughness when the applied load equals the ultimate stress of $P=60\%\;\sigma_{max}$. Creep strain for a longitudinal (**a**) and transverse (**b**) surface texture mode in sensitive grids; (**c**) creep strain for the side-wall profile of sensitive grids.

Choosing 60% of the ultimate working stress ($P=60\%\;\sigma_{max}$) as the load, we calculated the creep strain of the sensitive grid models to obtain the curves in Figure 8. It should be noted that, since the creep strain represents the relative elongation of the material, we use the number 1 to represent its unit. Based on Norton–Bailey creep theory, only the first and steady-state stages of creep strain were investigated.

**Figure 9:**
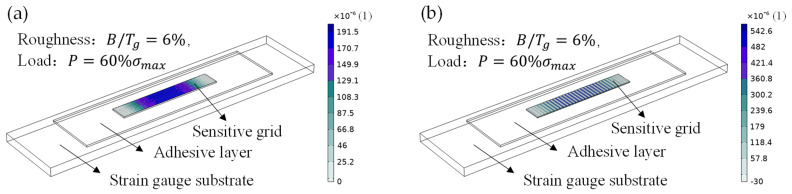
Creep strain distribution for a longitudinal (**a**) and transverse (**b**) profile mode of sensitive grids.

The result shows that the creep strain increases with the roughness. In Figure 8a, there is a sharp increase when the roughness of longitudinal textures ($B/T_{g}$) is greater than 6%, indicating that stress concentrations exacerbate greatly after accumulating to a certain amount [6,7]. In Figure 8b, the creep strain curves increase moderately with the roughness of transverse textures. Furthermore, the creep strain caused by the side-wall profile roughness shown in Figure 8c is increased by one order of magnitude compared with the former two. Clearly, at the same roughness, the creep strain caused by longitudinal textures is less than that of the transverse. In other words, the creep effect led by longitudinal textures is less obvious when strain gauges are subjected to same amount of resistance trimming. The cloud diagram of creep strain distribution shown in Figure 9 also verified this conclusion: the creep strain is much smaller when the direction of textures is consistent with the length direction of sensitive grids.

**Figure 10:**
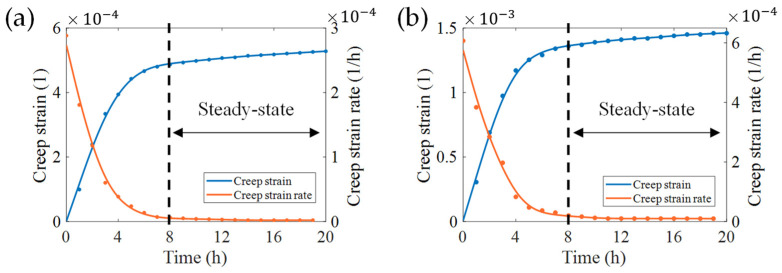
Creep and creep rate for a transverse surface profile (**a**) and side-wall profile (**b**) of sensitive grids. The creep curve is the same as the condition of $B/T_{g}=8\%$ shown in Figure 8b, $A/W_{g}=8\%$ shown in Figure 8c. The creep rate approaches to zero beyond 8 h.

We used derivatives to determine the point at which the curves in Figure 8 enter the steady-state. Considering the greater creep effect caused by the transverse textures and the side-wall profile described previously, we pay extra attention to the moment when the creep strain enters the steady-state in the both cases. It can be observed that the two curves in Figure 8b,c with $A/W_{g}=B/T_{g}=$ 8% tend to the steady-state somewhat more slowly compared to the others, which means that, by the time these two curves enter the steady-state, so have the curves for the other roughness conditions. Therefore, two curves, $B/T_{g}=8\%$ in Figure 8b and $A/W_{g}=8\%$ in Figure 8c, were selected to solve the creep strain rate (Figure 10). In this paper, we define: the creep strain enters to steady-state when the strain satisfies:

(2) $$\left|\dot{\varepsilon_{i}}-\dot{\varepsilon_{i+1}}\right|/\dot{\varepsilon_{i}}<18\%\;,\;\left(i=0,1,2,\ldots ,20\;h\right),\;$$

We can see that there are two trends presented in Figure 10a,b: the rate decreases rapidly at the beginning and maintains a constant approaching to zero later. In fact, the creep strain at the adjacent sampling points conforms to Equation (2) at the 8th hour where creep enters a steady-state. These trends are also in accordance with the first two stages of a typical creep strain curve.

When the perfect flat models (A = B = 0) are simulated, the creep strain curves acquired are approximately the same, and the average creep strain in steady-state (8–20 h) is about $2.40\times 10^{-4}$. However, this value of the three models with maximum roughness is 1.89, 3.12, and 6.95 times that of the perfect flat models, respectively. The creep strain that resulted from the side-wall profile is far greater than that of the surface textures.

**Figure 11:**
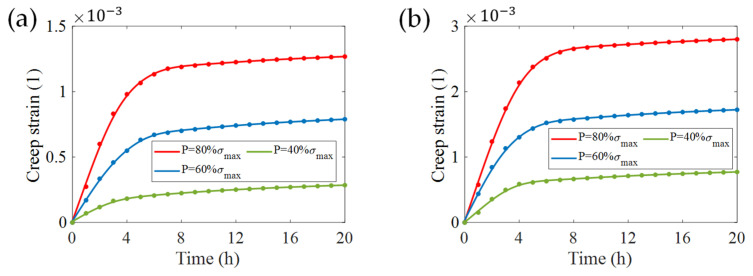
Creep strain induced by varying stress load for (**a**) transverse textures and (**b**) the side-wall profile of sensitive grids in the conditions of with $B/T_{g}=8\%$ (**a**) and with $A/W_{g}=8\%\;$ (**b**).

Under the conditions of maximum roughness, the effects of the load magnitude on creep strain were analyzed. According to the achievements from Figure 8, we only considered situations in the roughness of transverse textures and side-wall profile. As shown in Figure 11, curves still follow the creep law of the materials, and the creep strain increases in proportion to the load. Numerically speaking, under the same load conditions, the creep strain caused by side-wall profile is about twice that of the transverse textures. This phenomenon also tallies with the conclusion in the previous paragraph.

### 3.2. Fatigue Life

**Figure 12:**
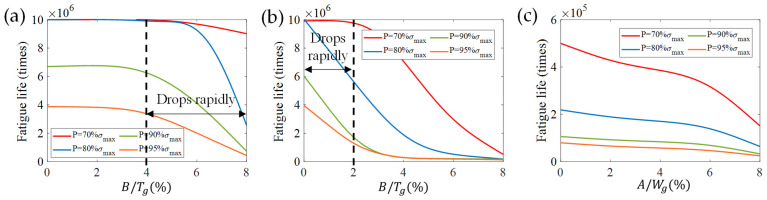
Fatigue life of sensitive grids for different surface roughness (**a,b**) and side-wall profile (**c**) under varying stress loading. (**a**) longitudinal, (**b**) transverse surface texture mode, and (**c**) side-wall profile of sensitive grids.

Load is a major contributing factor in the fatigue life of material. In this paper, we also investigated the fatigue life of the three models in different surface roughness and varying working stress conditions. According to the S–N curve (Figure 7b), the fatigue life was calculated as shown in Figure 12.

Two obvious features are shown in Figure 12a,b: on the one hand, with the applied load increases from 60% of the ultimate stress to 95%, the fatigue life decreases sequentially; on the other hand, the fatigue life reduces along with the growth of the surface roughness. When the sensitive grids are subjected to the high-strength load (defined as 80, 90, 95% of the ultimate stress), there is a rapid decrease in fatigue life as $B/T_{g}$ gradually increases from 0 to 2% as shown in Figure 12b. As for Figure 12a, only when $B/T_{g}$ is greater than 4% does the fatigue life start to drop quickly—meaning that, when the roughness is the same, the longitudinal textures have a smaller effect on fatigue life. Considering the phenomena presented in Figure 8a,b, the strain gauges have lower creep strain and longer fatigue life, when the textures go along with the length direction of sensitive grids. In addition, compared with Figure 12a,b, the fatigue life is reduced by an order of magnitude in Figure 12c. Under the condition of loading 70% ultimate stress, the fatigue life reaches 4.1 × 10^5^ times when $A/W_{g}\;$= 2% (Figure 12c) and 10^7^ times when $B/T_{g}$ = 2% (Figure 12a,b), and the latter is 25 times of the former. As a result, optimizing the wet etching parameters and reducing the peak/valley values of the side-wall profile can prolong the fatigue life of strain gauges.

## 4. Conclusions

In this paper, the effects of surface textures and side-wall roughness on creep strain and fatigue life for strain gauge were investigated. We observed the surface micro-morphology of commercial sensitive grids by microscope, and analyzed their range of geometric dimensions. Based on the observation results, three equivalent sinusoidal geometric models for the upper surface and side-wall profile were established. By using Norton–Bailey time-hardening theory and S–N curve, we analyzed these models and summarized the dependence of the micro-morphology caused by wet etching and resistance trimming on creep behavior and fatigue life.

Creep strain and fatigue life are affected by the roughness of surface textures and side-wall profile. Both would lead to the increase of creep strain and the decrease of fatigue life in varying degrees. Compared with the longitudinal textures, the transverse ones affect creep strain and fatigue life more obviously. Among the three models, the side-wall profile roughness has the greatest effect on the creep strain and fatigue life: 1. The creep strain led by the side-wall profile model is increased by one order of magnitude compared with the other two; 2. The steady-state average strain calculated by the maximum roughness of the side-wall profile is up to 6.95 times that of the perfect flat surface; 3. Under the conditions of same roughness and working stress, the side-wall profile can lead to the reduction of fatigue life to 1/25 of the textured surface.

Research findings indicate that the resistive strain gauges follow the laws of creep behavior and fatigue life for classical metal materials. However, the effects of different surface textures and side-wall profile on the creep behavior and fatigue life are different. The obtained achievements promote an understanding for optimizing the fabrication process of resistive strain gauges. The side-wall profile of sensitive grids has the most significant effect on creep behavior and fatigue life, so it is necessary to optimize the wet etching process of constantan alloy thin film to reduce the peak/valley values of the side-wall profile. As for resistance trimming, strain gauges should be polished along with the length direction of sensitive grids so as to achieve low creep strain and long fatigue life. In the follow-up research, we will continue to study the effects of temperature and thickness variation of the side-wall edges on creep law. Furthermore, experimental test and analysis will be carried out to clarify the mechanism of the micro-morphology on the creep behavior and fatigue life.

## Figures and Tables

**Table 1:** Basic material parameters of three layers.

Layer of Model	Material	Poisson’s Ratio	Young’s Modulus (Pa)
Sensitive grid	Constantan alloy	0.329	$1.6\times 10^{11}$
Adhesive layer	Epoxy resin	0.38	$5\times 10^{9}$
Strain gauge substrate	Polyimide	0.272	$1.96\times 10^{11}$

**Table 2:** Size data of model (unit: μm).

$L_{g}$	$L_{a}$	$L_{b}$	$W_{g}$	$W_{a}$	$W_{b}$	$T_{g}$	$T_{a}$	$T_{b}$
300	600	850	50	150	200	5	5	25

**Table 3:** Creep constants of constantan alloy.

Creep Rate Coefficient *C* (1/h)	Stress Exponent *n* (MPa)	Time Hardening Exponent *m*
3.125 × 10^–14^	1	0.5

## Data Availability

This study did not report any data.

## Supplementary Materials

The following are available online at https://www.mdpi.com/article/10.3390/mi13030379/s1, Figure S1: Creep curve (a) and creep rate curve (b) of general metal elastic materials, Figure S2: (a) Step load applied for the creep simulation. (b) S-N curve of alloy steel used.
