# Supplementary material for: Dependence of Creep Strain and Fatigue Behavior on Surface Characteristics of Resistive Strain Gauges
Source: Micromachines (Basel). 2022 Feb 26;13(3):379. doi: 10.3390/mi13030379 (PMC8955004; doi:10.3390/mi13030379)
Supplement: Supplementary file 1 [file micromachines-13-00379-s001.zip › micromachines-1560926-supplementary.pdf]

micromachines

Supplementary Information: Dependence of Creep Strain and Fatigue Behavior on Surface Characteristics of Resistive Strain Gauges

Thesis for Creep Strain and Fatigue Life

The creep effect of metallic material refers to the process where the internal stress of the material varies with time and results in plastic deformation when the material is subjected to constant temperature and load [1]. In other words , in the condition of constant load and temperature, creep effect manifested as plastic deformation varies along with the changes of stress inside material. In fact, the creep behavior of resistive strain gauges affects the performance of long-term stability and repeatability of force sensors. And the creep strain appertains to the one only caused by creep effect, it is defined as the difference between total and elastic strain under external force.

When constant load is input, the typical creep (rate) curve of materials can be divided into three stages shown in Figure S1a and 1b. During the first stage (0-t_b), the creep increases rapidly over time while the creep rate decreases sharply. With the recrystallization of atoms in the material, dislocation and tangle of crystal lattice bring with the increase of density, so that the creep rate decreases to minimum eventually. The second stage of the creep curve is the steady-state. In this stage, the creep increases slowly, but the creep rate approximately maintains a constant. The stable working life of material is characterized by the time interval from t_c to t_b. The increment between the segment bc in Figure S1a represents the creep index of materials, and the creep rate ($\dot{\varepsilon_b}$) of an ideal material approaches zero. In general, the second stage of creep is regarded as the main part of entire creep life. While strain gauges are working in this stage, their inner strains are mainly manifested as recoverable elastic ones, which are the elastic properties of the constantan alloy.

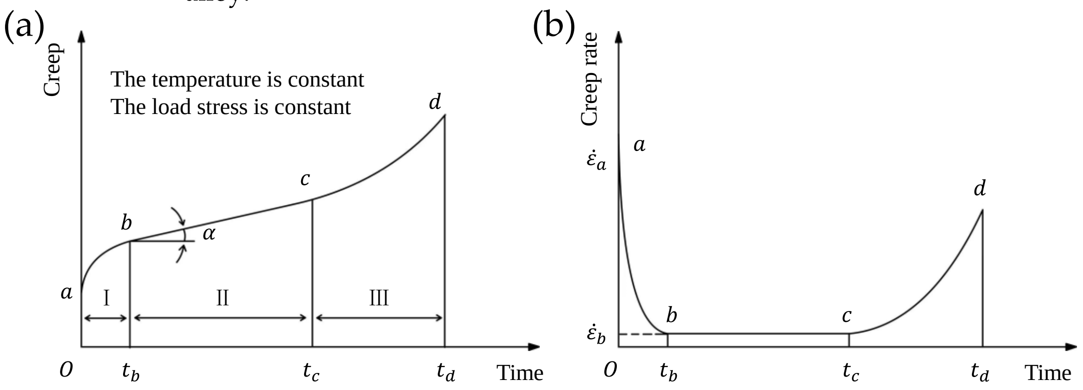

Figure S1. Creep curve (a) and creep rate curve (b) of general metal elastic materials [1].

Creep enters an accelerating phase accompanied with the aggravation of damage. In the third stage of the creep curve, creep rate increases rapidly to the strength limit point d of the material [1]. Generally, when mechanisms exceed their expected life span due to repeated use, the creep stress inside the material will increase sharply as the transition from micro-holes and micro-cracks among the lattice to macro-cracks occurs. Moreover, the interval between time points t_c and t_d is usually unpredictable. As a result, creep behavior of resistive strain gauges in the first and second stages is our main study task.

The creep strain of material is mainly related to time, temperature and stress on the material [2]. Norton-Bailey time-hardening theory assumes that under the condition of constant load and temperature, creep strain is only a function of time but no relevance with other factors.

Strain exponential equation based on this assumption is [3]:

$$\varepsilon_c = A\sigma^n t^m, \quad (1)$$

Creep rate can be acquired by differentiating the formula (1):

$$\dot{\varepsilon_c} = mA\sigma^n t^{m-1}, \quad (2)$$

In formula (2), parameters A, n and m remain constant at a certain temperature, they can be measured with experiment. When m<1, the creep rate expressed by formula (2) decreases over time, and remains constant when m=1. This equation, called time-hardening function, can describe the first stage (when m<1) and stable-state (when m=1) of the creep curve shown in Figure S1 [3].

A common deformation process of metal materials subjected to external force consists of three stages: elastic, plastic and instability deformation. Elastic deformation refers to the linear positive correlation between material deformation and stress, and the deformation is completely restored after the stress is removed. Plastic deformation refers to the residual deformation after stress removal when the stress is beyond the yield limit of the material. Instability deformation means that when the stress continues to increase, the metal structure will fracture due to a large amount of plastic deformation [4,5]. Still, when the structure is subjected to repeated varying loads, fatigue damage may occur even the stress does not exceed the strength limit or is lower than the elastic limit of the material. Fatigue refers to the process that permanent deformation, such as local cracks or complete fracture, happens to solid materials due to sufficient external cyclic stress action [4,6]. Accordingly, fatigue life refers to the number of cycles of fluctuating stress that a component can withstand before the plastic crack and fracture failure appear. The fatigue life, reflecting the ability of materials to resist fatigue damage, depends not only on the loading stress, but on the loading time and frequency. Here, loading stress includes periodic cyclic loads and irregular random ones. In actual application, resistive strain gauges used in force sensors are mainly subjected to the former. Also, the material of the resistive strain gauges we discussed works at the low-stress fatigue stage which is in the range of elastic deformation.

Fatigue behavior of resistive strain gauges directly affects the reliability and working life of force sensors. The fatigue curve (also called S-N curve), generally given in the form of double logarithms ($\log \sigma_a$-$\log N_f$), describes the correlation between the value of stress σ_a and the number of cycles N_f when fatigue damage occurred in metal structure [7-9]. The fatigue life can be regarded as infinite when the material is subjected to low-stress. As the stress increases continuously, there is a linear relation between $\log \sigma_a$ and $\log N_f$ in a certain range depended on the types of materials. However, no mathematical expression is suitable for S-N curve where the stress amplitude approaches the fatigue limit [6,7].

The cyclic stress can be classified as uniaxial stress and multiaxial one, and strain gauges work in the range of uniaxial elastic deformation process. The uniaxial cyclic stress ratio is defined as the ratio of the minimum to the maximum of external stress. The fatigue life will decrease while the absolute value of the stress ratio does oppositely. Suppose the minimum and the maximum of the external stress are equal in value, opposite in sign, the shortest fatigue life of the structure can be obtained. In addition, the S-N curve of a certain material is not fixed, but is also related to the stress ratio. It is impossible to calculate the S-N curve at each stress ratio in practice since there are infinite situations. In order to simplify the research process, the minimum external stress of the strain gauge in this study is set to zero. Under this condition, the fatigue life obtained is the maximum one.

For the creep and fatigue are both dynamic processes, the transient was selected for all simulations. Creep simulation is carried out under a constant load as shown in Figure S2a. The load value was 60% of the ultimate stress of the strain gauge, and the loading time was 20 hours. During fatigue simulation, the S-N curve of alloy steel was settled as the reference criteria shown in Figure S2b. Under different loads, we simulate the stress distribution of the strain gauge; Calculate the maximum internal stress value in this distribution; Then set this maximun value as the ordinate in the S-N curve and we can get the corresponding abscissa which refers to the fatigue life.

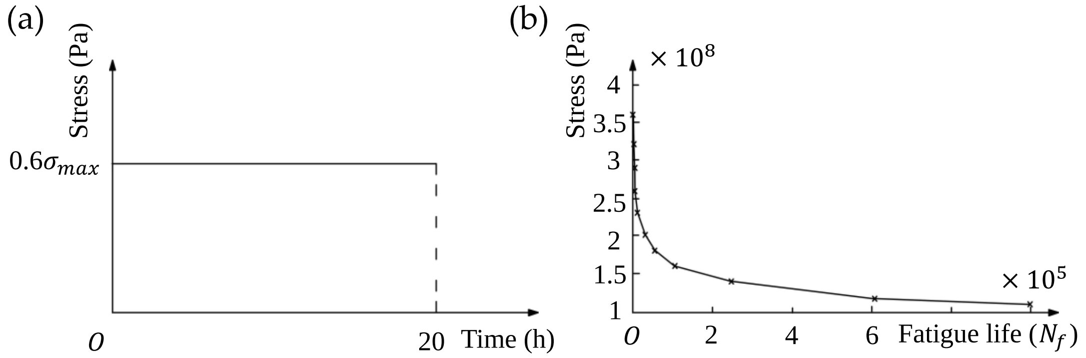

Figure S2. (a) Step load applied for the creep simulation. (b) S-N curve of alloy steel used.

Reference

1. Delin Shu. Mechanical Properties of Engineering Materials[M]. Beijing, China Machine Press, **2016**.04. (in Chinese)
2. El-Nahass M M, Zeyada H M, Aziz M S, et al. Optical Properties of Thermally Evaporated SnS Thin Films[J]. Optical Materials, **2002**, 20(3): 159-170. [CorssRef]
3. Sanchez-Juarez A, Tiburcio-Silver A, Ortiz A. Fabrication of SnS2/SnS Heterojunction Thin Film Diodes by Plasma-enhanced Chemical Vapor Deposition[J]. Thin Solid Films, **2005**, 480: 452-456. [CrossRef]
4. Chuanyue Zhou, Hongxia Zheng, Huiqiang Luo, et al. MSC. Fatigue application and examples of fatigue analysis[M]. Beijing, Science Press, **2005**.03. (in Chinese)
5. Jianpei Zhao. Research on Theoretical Forming Limit Diagram of AA5083 Thin-Shell at Elevated Temperature[D]. Yanshan University, **2016**. (in Chinese)
6. Chuanyao Chen. Fatigue and Fracture[M]. Wuhan, Huazhong University of Science & Technology Press, **2002**. (in Chinese)
7. Shanqin Hou. Life Evaluation Model of Stress Fatigue for Metals[D]. Shanghai Jiao Tong University, **2016**. (in Chinese)
8. Chaotao Liu. Fatigue Simulation Analysis of Notched Specimen Based on Master S-N Curve. Dalian Jiaotong University, **2014**. (in Chinese)
9. Dong P, Hong J K, Osage D A, et al. Master SN Curve Method for Fatigue Evaluation of Welded Components[J]. Welding Research Council Bulletin, **2002** (474). [CorssRef]
